# *In Silico*, *In Vitro* and *In Vivo* Analysis of Binding Affinity between N and C-Domains of *Clostridium perfringens* Alpha Toxin

**DOI:** 10.1371/journal.pone.0082024

**Published:** 2013-12-11

**Authors:** Siva Ramakrishna Uppalapati, Joseph Jeyabalaji Kingston, Insaf Ahmed Qureshi, Harishchandra Sripathy Murali, Harsh Vardhan Batra

**Affiliations:** 1 Microbiology Division, Defence Food Research Laboratory, Mysore, Karnataka, India; 2 Department of Biotechnology, School of Life Sciences, University of Hyderabad, Hyderabad, India; Institute Pasteur, France

## Abstract

*Clostridium perfringens* alpha toxin/phospholipase C (CP-PLC) is one of the most potent bacterial toxins known to cause soft tissue infections like gas gangrene in humans and animals. It is the first bacterial toxin demonstrated to be an enzyme with phospholipase, sphingomyelinase and lecithinase activities. The toxin is comprised of an enzymatic N-domain and a binding C-domain interconnected by a flexible linker. The N-domain alone is non-toxic to mammalian cells, but incubation with C-domain restores the toxicity, the mechanism of which is still not elucidated. The objectives of the current study were to investigate the formation of a stable N and C-domain complex, to determine possible interactions between the two domains *in silico* and to characterize the *in vitro* and *in vivo* correlates of the interaction. To establish the existence of a stable N and C-domain hybrid, *in vitro* pull down assay and dot-Far Western blotting assays were employed, where it was clearly revealed that the two domains bound to each other to form an intermediate. Using bioinformatics tools like MetaPPISP, PatchDock and FireDock, we predicted that the two domains may interact with each other through electrostatic interactions between at least six pairs of amino acids. This N and C-domains interacted with each other in 1:1 ratio and the hybrid lysed mouse erythrocytes in a slower kinetics when compared with wild type native Cp-PLC. BALB/c mice when challenged with N and C-domain hybrid demonstrated severe myonecrosis at the site of injection while no death was observed. Our results provide further insight into better understanding the mechanism for the toxicity of Cp-PLC N and C-domain mixture.

## Introduction

*Clostridium perfringens* is a Gram-positive, spore-forming, obligate anaerobe commonly found in the gastrointestinal tracts of both humans and animals, and in soil, food and sewage [1,2]. Although it is a part of normal flora of the intestine; due to occasional dietary stress, extensive antibiotic usage, injury or any other favourable conditions, this pathogen causes some of the dangerous maladies; *viz*., necrotic enteritis, enterotoxemia, enteritis necroticans (pigbel), gas gangrene (clostridial myonecrosis) etc [2]. These disease manifestations can be attributed to the ability of *C. perfringens* to produce a plethora of invasins and exotoxins. The organism is categorized into five toxinotypes (A-E) based on the production of four major lethal toxins (alpha, beta, epsilon and iota) [3]. Alpha toxin, produced by all toxinotypes of *C. perfringens*, is the chief virulence factor known to be involved in the necrotic enteritis and gas gangrene disease, a condition where necrotizing tissue is associated with gas production [4].

*C. perfringens* alpha toxin or phospholipase C (Cp-PLC) is the first bacterial toxin demonstrated to be an enzyme [5]. The toxin possesses lecithinase, phospholipase and sphingomyelinase activities that result in haemolysis and necrosis [4]. It is a zinc metalloenzyme that can bind to membranes in the presence of calcium ions [6]. Encoded by a chromosomal borne plc gene, Cp-PLC is a 398 amino acid holoprotein with an N-terminal signal sequence of 28 amino acids, which when cleaved results in a mature protein of 43 kDa [7]. The haemolytic and necrotic property of the toxin is demonstrated to be mediated via activation of the sphingomyelin metabolic system through GTP-binding proteins or through the activation of glycerophospholipid metabolism [8]. The toxin also induces carboxyfluorescein leakage and phosphorylcholine release from liposomes [4] and neuritogenesis in pheochromocytomal PC12 cells [9]. 

Crystallographic studies revealed that Cp-PLC had two domains: the nine-alpha helical N-domain (similar to *Bacillus cereus* PLC) and an eight-stranded antiparallel β-sandwich C-domain (similar to C2 domains of eukaryotic proteins). A ten amino acid flexible linker (residues 247–255: GNDPSVGKNV) connects the two domains [6]. The alpha toxin N-domain is catalytic and the C-domain is involved in binding the toxin to biological membranes. The N-domain possesses phospholipase activity but cannot induce hemolysis in the absence of C-domain and mixing the individual N-domain and C-domain restores the hemolytic activity [10,11]. The mechanism for this restoration is not yet elucidated. In the present study, *in silico, in vitro* and *in vivo* approaches were followed for bioinformatics prediction and experimental validation of the interactions between N and C-domains of *C. perfringens* alpha toxin, respectively. We identified that the restoration of the haemolytic and myonecrotic property of N and C-domain mixture is mediated via formation of a functional intermediate in the solution. Following *in silico* analysis, we identified that the N and C-domain hybrid is stabilized through electrostatic bonds between at least six pairs of amino acids. 

## Materials and Methods

### Bacterial strains, Plasmids and Media

*C. perfringens* ATCC 13124 was supplied by American Type Culture Collection, USA. *E. coli* DH5α and BL21 DE3 strains and pRSET A plasmid were procured from Invitrogen, Bengaluru, India. All the media were procured from Himedia (Mumbai, India). *C. perfringens* culture was maintained in Fluid Thioglycollate broth and incubated in anaerobic jars (Himedia) at 37° C. Luria Bertani broth with appropriate amount of ampicillin antibiotic (Sigma, India) was used in all cloning and expression experiments.

### *In silico* analysis of interactions between N and C-domains of *C. perfringens* PLC

Prediction of protein-protein interaction sites was done using MetaPPISP [12], while protein-protein docking was performed with the web version of PatchDock [13] and then further refined and ranked with FireDock [14]. The crystal structure of Cp-PLC (PDB ID: 1CA1) was retrieved from Protein Data Bank. Since the toxin contains two domains are interconnected with a linker, both were separated and used for docking study with N-terminal domain as receptor and C-terminal domain as ligand under default complex-type settings. Molecular visualization and general analysis were done using the program PyMOL [15]. HBOND program was used to identify hydrogen bonds at the molecular interface [16]. 

### Cloning, expression and purification of recombinant N and C-domains of PLC

The genes corresponding to N and C-domains of *C. perfringens* PLC (excluding the ten amino acid linker) were PCR amplified using the primer pairs NF (5’ CTCGAGTTGGGATGGAAAGATTGATG 3’), NR (5’ AAGCTTTCTGATACATCGTGTAAGAATC 3’) and CF (5’ CTCGAGAAAAGAACTAGTAGCTTACAT 3’), CR (5’ AAGCTTATTTTATATTATAAGTTGAA 3’), respectively. The primer pairs had overhangs of restriction sites for *Xho* I (underlined in both NF and CF primers) and *Hind* III (underlined in both NR and CR primers) at their 5’ ends. The N and C-domain amplicons and pRSET A vector were digested with corresponding restriction enzymes (NEB, UK) and ligated together to form pRSET A-rN and pRSET A-rC recombinant vectors. The recombinant vectors were transformed into *E. coli* DH5α and clones were screened by PCR using universal T7 primers. Recombinant plasmids were extracted from PCR-positive clones and the insert genes were sequenced (Eurofins, India). Plasmids with in-frame inserts were transformed into *E. coli* BL21 DE3. Overnight cultures of transformants were re-inoculated into fresh LB broth (1:100) and incubated at 37 °C with shaking until an OD of 0.6 was reached. The rN and rC clones were induced with 1 mmol l^-1^ Isopropyl β-D-thiogalactopyranoside (Sigma, India) for 5 h. Bacteria were harvested by centrifugation at 10,000 *g* for 10 min, resupended in 1/10^th^ volume PBS (pH 7.4) and analysed for expression by SDS-PAGE analysis [17]. Expression positive clones were induced and the recombinant proteins, rN and rC were purified in non-denaturing conditions by immobilized metal affinity chromatography using Ni^2+^-NTA slurry (Qiagen, Germany) using buffers containing varying concentrations of Imidazole (Qiaexpressionist, Qiagen, Germany). The fusion proteins were treated with enterokinase enzyme and the truncated proteins were purified by enterokinase cleavage capture kit (Invitrogen, Bengaluru, India) as per manufacturer’s protocol. The purified recombinant proteins were dialysed against PBS overnight at 4 °C; ascertained for purity by SDS-PAGE and quantified by Bradford’s method against known bovine serum albumin standards. The antigenicity of rN and rC proteins were analysed by Western blot analysis using anti-PLC polysera (Sigma, India) as probe.

### Immunization

Specific-pathogen-free (SPF) female BALB⁄c mice (procured from Central Animal Facility, D.F.R.L., Mysore, India) that were provided with food and water *ad libitum* were injected with 50 µg of rN antigen in Freund’s complete and incomplete adjuvant through intramuscular route at 10-day intervals for a total of four doses. Polyclonal antisera were separated from the blood collected through retro-orbital sinus and stored in 20 °C until further use.

### *In vitro* protein pull down assay

To test for the physical interactions between rN and rC-domains, pull down assay was performed [18]. Fifty microgram 6x His tagged rC-domain was added to equal amount of enterokinase treated rN-domain and incubated for at least 1 h at room temperature on a shaker. The protein solution was mixed with 50 µl Ni^2+^ NTA slurry and incubated for 2 h on shaker at 4 °C followed by washing the slurry thrice with PBS (pH 7.4). After washing, the adsorbed proteins were eluted into 50 µl Laemlli buffer for SDS-PAGE analysis. 

### Far-dot ELISA

To evaluate the affinity and specificity of binding of rN domain to rC-domain, Far-dot ELISA technique was preformed according to Ohba et al. [19] with minor modifications. Briefly, recombinant C-domain was spotted onto Nitrocellulose membrane and the membrane was treated with 5% skim milk solution for 1 h to saturate and block all the free binding sites. For probing, BSA-blocked membranes were incubated for 2 h with purified native rN-domain followed by reaction with anti-rN polysera raised in mouse. Reactivity of the anti-rN serum was visualized by addition of a goat anti-mouse IgG-Horse radish peroxidase conjugate and substrate 3,3’,5,5’-Diaminobenzidine tetrahydrochloride. All the washing steps were performed with PBST (PBS + 0.1%Tween 20).

### *In vitro* protein complementation assay

Protein complementation assay was performed to assess the functional correlates of the interactions between rN and rC-domains i.e. haemolytic property of the N and C-domain hybrid protein. Mouse erythrocyte (mRBC) suspensions used in the study were prepared according to Uppalapati et al. [20]. Twenty micro moles of rN and rC-domains, individually or in combination and equal moles of native wild type alpha toxin were added to individual wells on mouse blood agar plates and incubated at 37 °C for 4h. Alternatively 10 micro moles each of the proteins were diluted to a final volume of 100 µl in microtiter wells with K-PBSA buffer (150 mmol l^-1^, NaCl, 20 mmol l^-1^, KH_2_PO_4_, pH-7.4, with 1% bovine serum albumin) and equal volume of 5% mouse erythrocytes were added. The extent of haemolysis in each microtiter well was indirectly measured after incubation at 37 °C for appropriate time by recording the absorbance of supernatant at 544 nm. The haemolytic units (H.U.) of wild type and N and C-domain hybrid were calculated following microtiter method using 50% mouse erythrocytes. One H.U. is defined as the amount of toxin/proteins required to increase the absorbance of the supernatant of 50% mRBC solution by 1.

### *In vivo* challenge

All animal experiments were approved by institutional Animal Ethical Committee at Defence Food Research Laboratory (D.F.R.L.), Mysore, India. Specific-pathogen-free (SPF) four week old female BALB⁄c mice (procured from Central Animal Facility, D.F.R.L., Mysore, India) and were provided with food and water *ad libitum*. Animals (#6 per group) acclimatized to laboratory received intramuscular injections of either 50 μg C-domain alone or 50 μg N-domain alone or 10 H.U. N+C-domain mixture or 10 H.U. wild type toxin. All the animals were kept under observation for 8 days and surviving animals were later euthanized by cervical dislocation. Periodically, representative animals from each of the groups were sacrificed and muscle tissues at the site of injection were observed for histopathological damage. 

### Statistical analysis

All the analytical experiments in the study were conducted in triplicate and the values were given as means ± SD. Graphical illustrations were constructed using Microsoft Excel2007 (Microsoft Corp., MD, USA) and GraphPad software.

## Results

### Cp-PLC N domain binds to C domain

**Figure 1:**
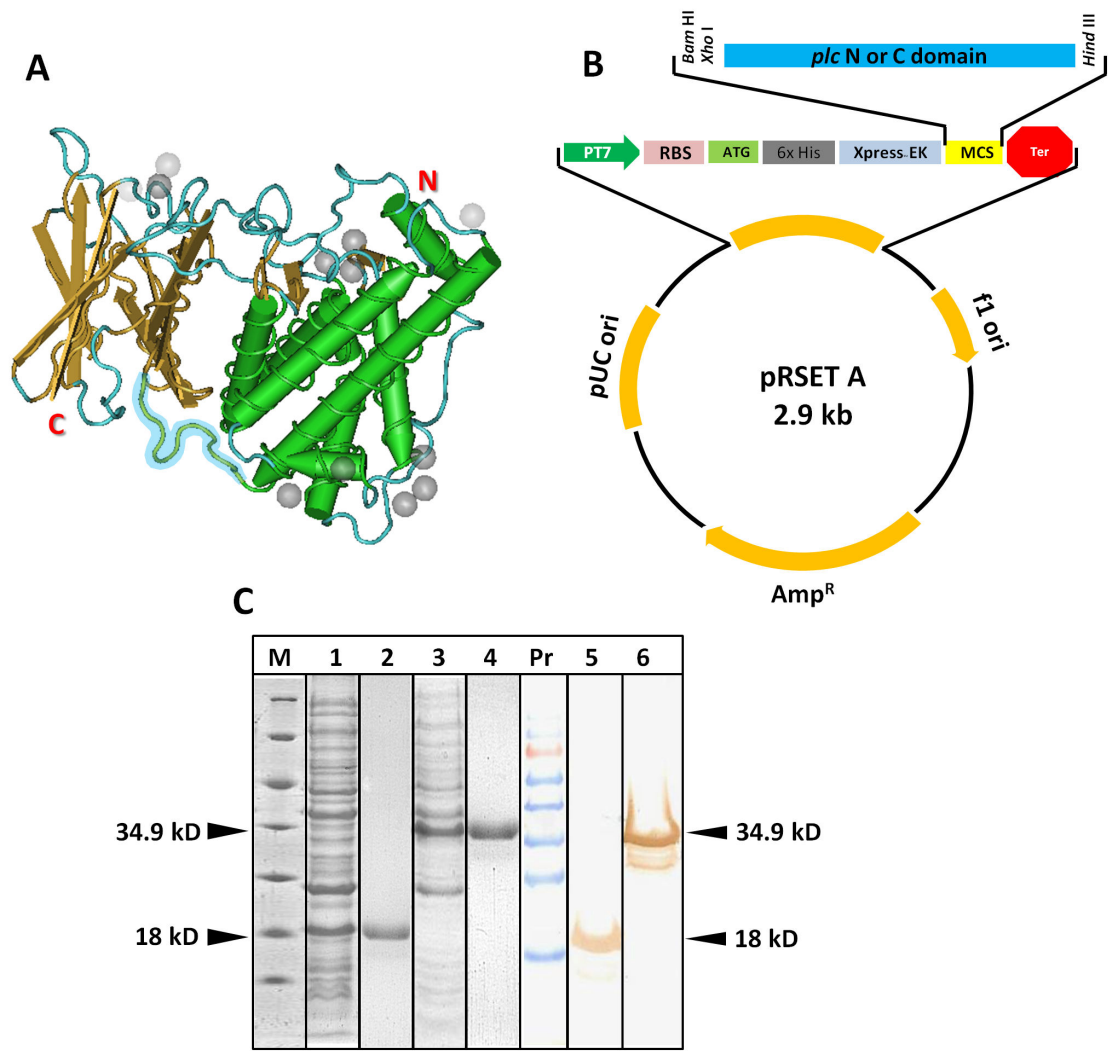
Construction of the recombinant expression vector pRSET A-PLC_N/C and cloning and expression of recombinant N and C-domains. (A) Crystal structure of *C. perfringens* phospholipase C protein (PDB ID 1MOX) drawn by Cn3D software. The ten amino acid linker GNDPSVGKNV region is highlighted in blue. (B) Schematic diagram of plasmid construct. Gene sequences corresponding to plc N or C-domain were inserted into pRSET A for the expression of recombinant protein. (C) Coomassie Blue stained 12% SDS-PAGE and western blot analysis of whole cell lysates of IPTG induced N and C-domain transformant *E. coli* cells. Arrow mark indicates the expressed recombinant proteins. Blot was probed with commercial anti-PLC polyclonal antibodies. M-Unstained protein marker; 1-induced recombinant C clone; 2- purified C-domain; 3-induced recombinant N clone; 4-purified N domain; PrPr-prestained protein ladder; 5-C-domain; 6-N-domain.

**Figure 2:**
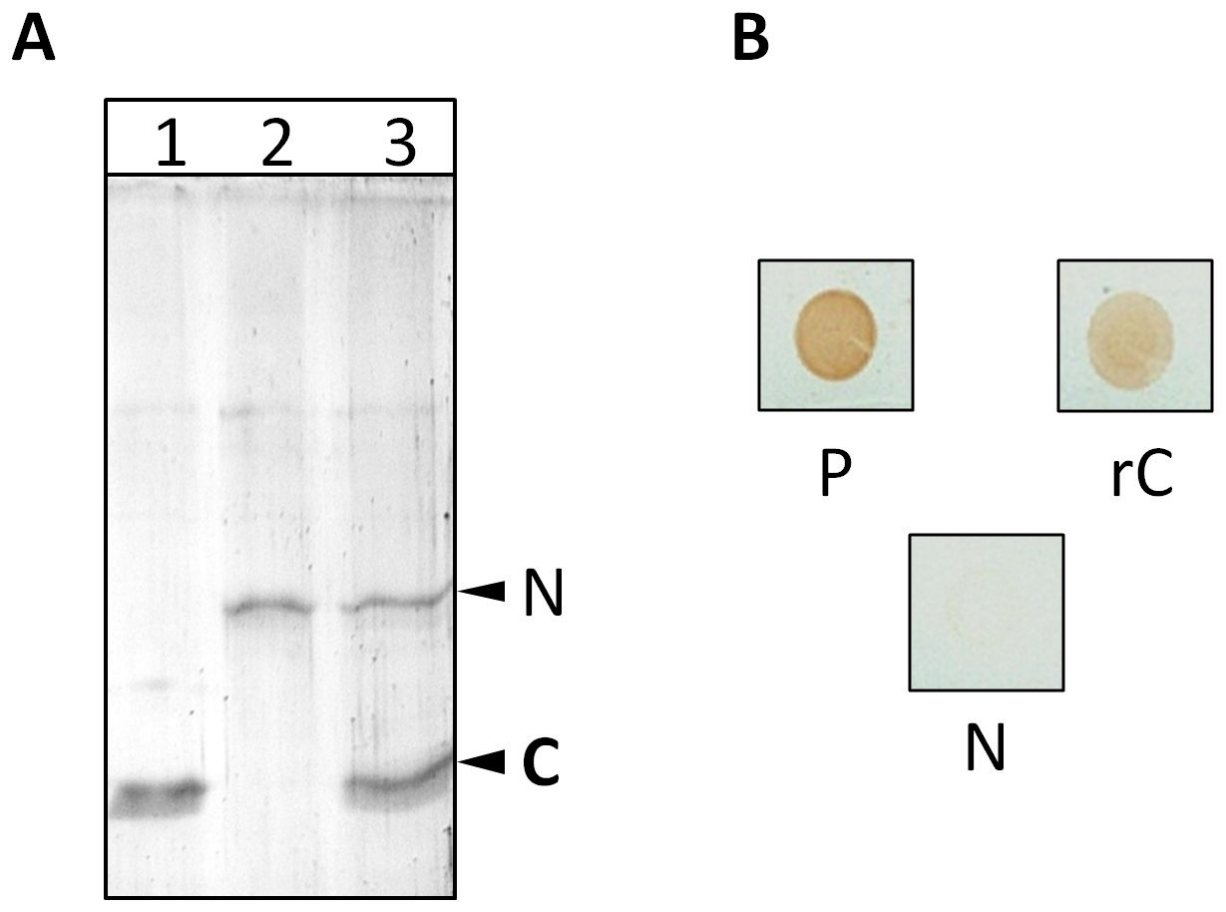
Cp-PLC rN-domain interacts with rC-domain. (A) Co-precipitation of N and C-domains. 12% SDS-PAGE analysis of purified rC (lane 1), rN (lane 2) and co-precipitated eluate of rC and rN (lane 3). The procedure was described in the main text. (B) Far-dot ELISA analysis of the interaction between rN and rC-domains. Nitrocellulose membranes containing spotted rC and rN (Positive control - P) and blocked with 5% milk solution were probed with rN and detected with mouse anti-rN primary antibodies. No immuno-reactive dot is present in PBS control (N).

The findings by Titball et al. [11] and Nagahama et al. [10] that the C-domain of *C. perfringens* alpha toxin complements the N-domain in restoring the hemolytic and cytolyitc activity prompted us to ask whether there is a stable protein-protein interaction between the two domains resulting in the formation of a N and C-domain hybrid. To establish the existence of such interaction, we cloned and expressed the two domains of Cp-PLC (Figure 1) and performed *in vitro* pull down and Far-dot ELISA assay. In the pull down assay, *in vitro* synthesized, recombinant N-domain (after enterokinase treatment) is allowed to interact with 6x His tagged C-domain. The N and C-domain hybrid is then precipitated by Ni-NTA slurry which binds only to 6x His tag. Figure 2A shows the SDS-PAGE profile of the eluates from the pull down assay which clearly indicated that N-domain and C-domain interacted with each other to form a stable hybrid. A non-specific protein like recombinant staphylococcal enterotoxin B (SEB) was also included in this experiment, to serve as negative control and no binding was observed between the Ni-NTA slurry and SEB. To estimate the strength of the interaction, we also asked whether the binding is affected by mild detergents, reasoning that if interaction involves weak bonds, the binding may be unstable. However, when Far-dot ELISA was carried out using mild detergent Tween 20 buffer for wash, the domains were still found intact, suggesting stable and strong interactions (Figure 2B). 

### Protein docking studies predicted stable interactions between individual N and C-domains

**Figure 3:**
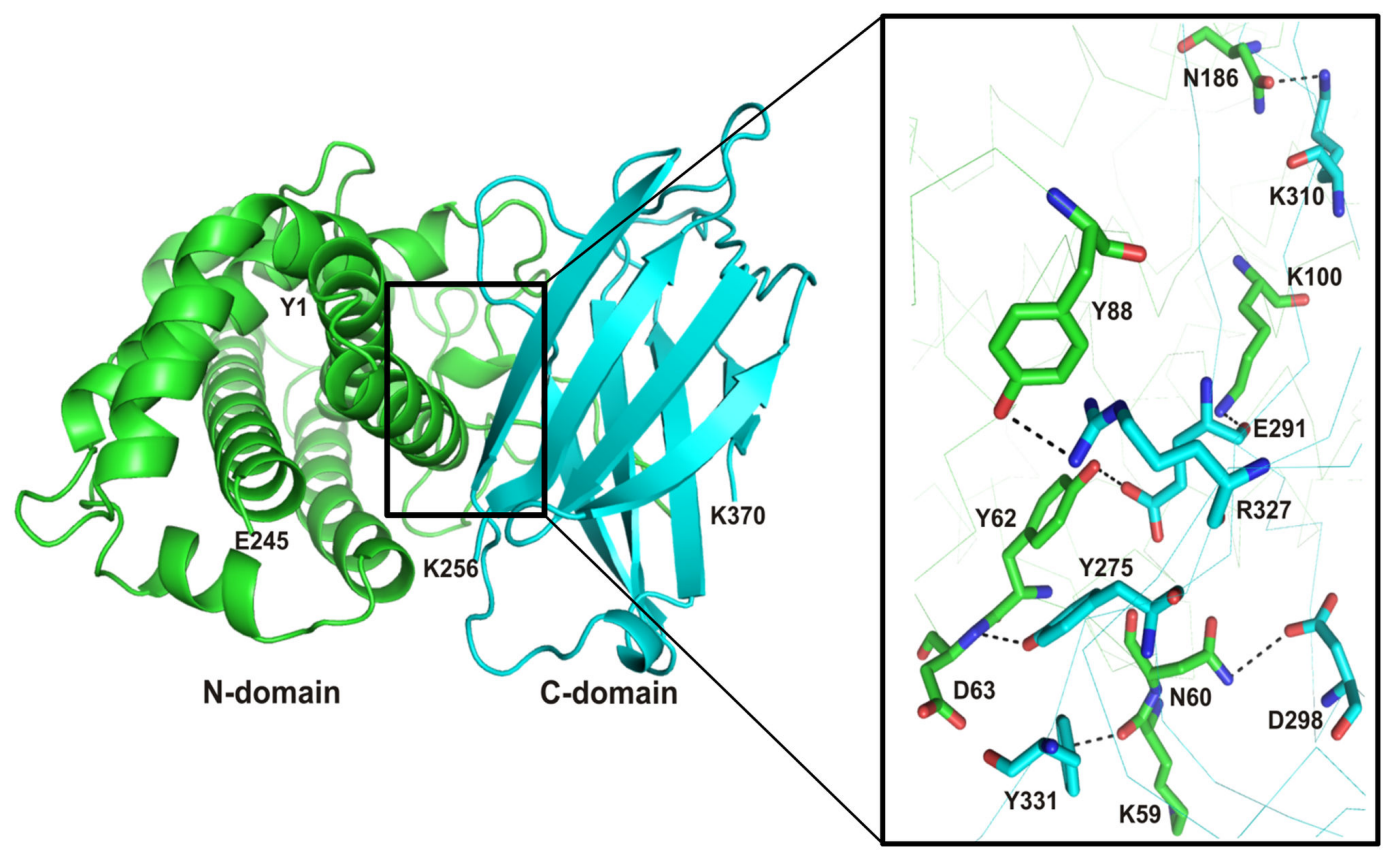
Putative interactions between N-terminal and C-terminal domain of *C. perfringens* alpha-toxin. The residues of N-domain and C-domain are colored in green and cyan respectively. The residues showing interaction between both proteins are labelled and displayed as stick model in element colors (carbon colored green/pink, nitrogen colored blue, and oxygen colored red). Hydrogen bonds are represented by black dashed lines.

**Figure 4:**
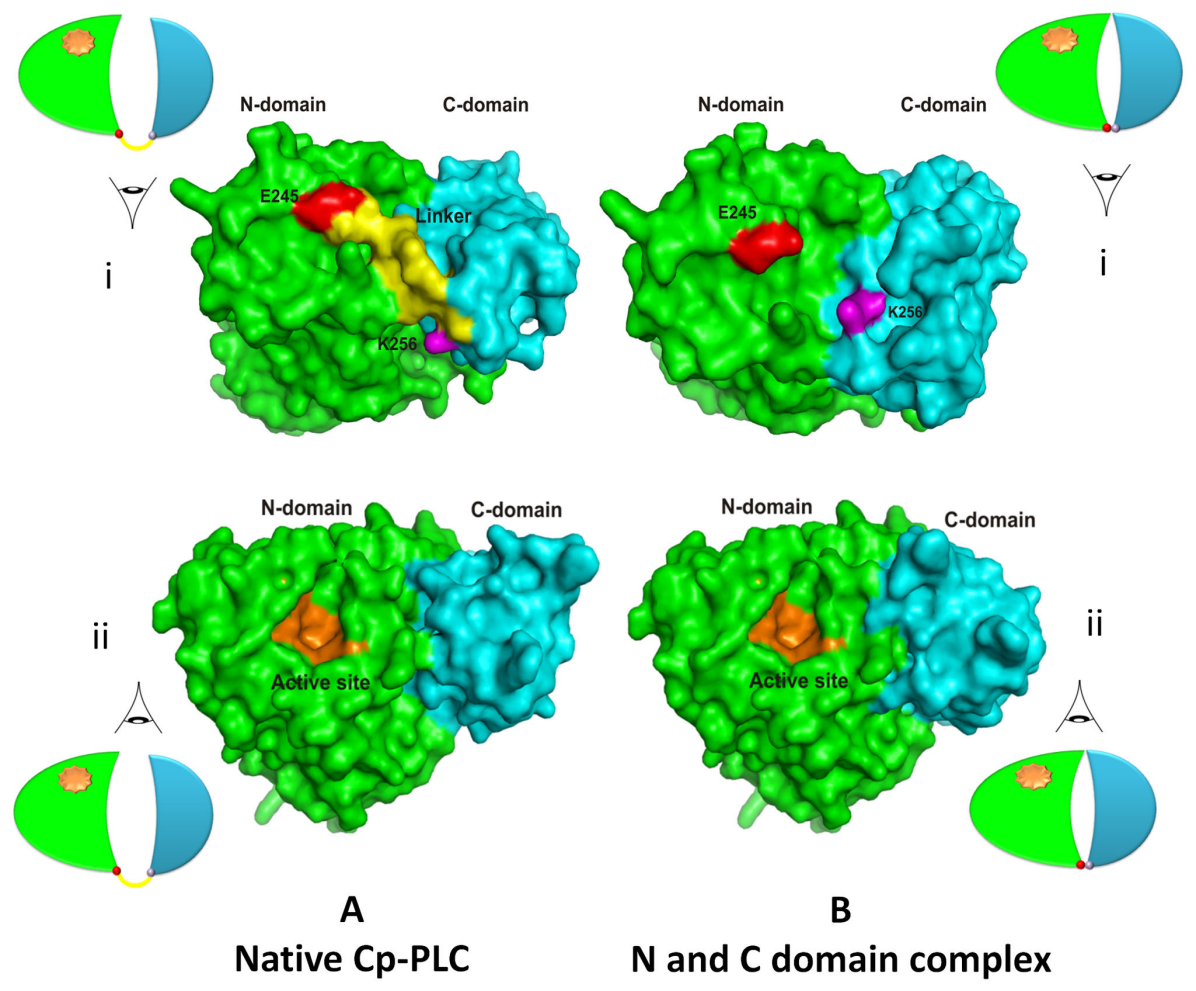
Surface representation of structures of native alpha toxin from *C. perfringens* and N and C-domain hybrid. A. Crystal structure of native alpha toxin from *C. perfringens* illustrating N and C-domains along with a linker (View i) and the active site (View ii). B. Docking study of N and C-domains presenting configuration changes during interaction (View i) and the unchanged active site on N domain (View ii). The N-domain and C-domain are colored in green and cyan respectively. The last residue of N and first residue of C are displayed in red and pink colors, respectively. The residues of linker region and active site are presented in yellow and orange colors, respectively.

Our next objective was to identify possible interactions between the N-terminal and C-terminal domains of Cp-PLC. Several programs such as HEX, GRAMM-X, Z-dock and PatchDock were employed for unbound protein-protein docking using N-domain as a receptor and C- domain as a ligand. Approximately, 1000 predictions were generated using PatchDock and all the predictions were submitted to FireDock to refine 10 best solutions on the basis of global energy. Several low-energy docking models emerging from this exercise placed the C-domain close to the N-domain. In the next step, we attempted to identify the interfacial area where these two domains are likely to bind without affecting the active site on N-domain. Mutation data from the previous studies showed that Asp 305 [21] and Tyr 307 [22] amino acids were located in the interfacial area of the two domains in the native toxin and mutations at these residues result in reduction of toxicity, thus delineating their role in toxicity. Based on this data, plausible interaction sites among the interface residues of both N and C-domains were chased by MetaPPISP. These residues were used to analyze the 10 docked complexes for the presence of such residues in the interface and most of the interfacial residues were present in the eighth ranked complex. Among all, one complex was found plausible based on the minimum energy score and binding interface residues (Figure 3). Other complexes contained C-domain binding at a different location on N-domain, and residues in this location were not predicted as interfacial residues by Meta-PPISP. Hence, these complexes were not taken into account for further analyses. The final docking model (Figure 3) shows N-domain binding with C-domain through interactions involving at least 6 pairs of amino acids (Table 1). In this model, no covalent bonds were observed, while all interactions were electrostatic in nature. In case of native wild type alpha toxin from *C. perfringens*, interaction between N-domain and C-domain is mediated via the linker (GNDPSVGKNV) that plays significant role in keeping both domains away from each other and to provide interim space for conformational changes induced upon membrane binding [6]. During protein-protein docking study in the predicted complex, it was observed that C-domain undergoes a minor change in spatial orientation while interacting with N-domain to form a complex and this slight shift was depicted in Figure S1. Despite the minor disorientation, the amino acids involved in Zn^2+^ ions on N-domain and Ca^2+^ ions on C-domain were found unaffected. In our predicted model, it is also evident that, no structural impediments or conformational changes are observed in the active site region on N-domain suggesting the unimpaired enzymatic activity (Figure 4).

**Table 1:** Putative residues involved in protein-protein interactions between N and C domains of *C. perfringens* Alpha-toxin found from meta-PPISP.

**N domain**	**C domain**	**Bond length (Å)**
D186	K310	2.5
D63	Y275	2.6
K59	Y331	2.6
Y62	E291	2.5
K100	E291	2.5
Y88	R327	3.2
N60	D268	3.3

### Cp-PLC N and C-domains bind to each other in 1:1 ratio and the hemolytic activity of the complex follows delayed type kinetics

**Figure 5:**
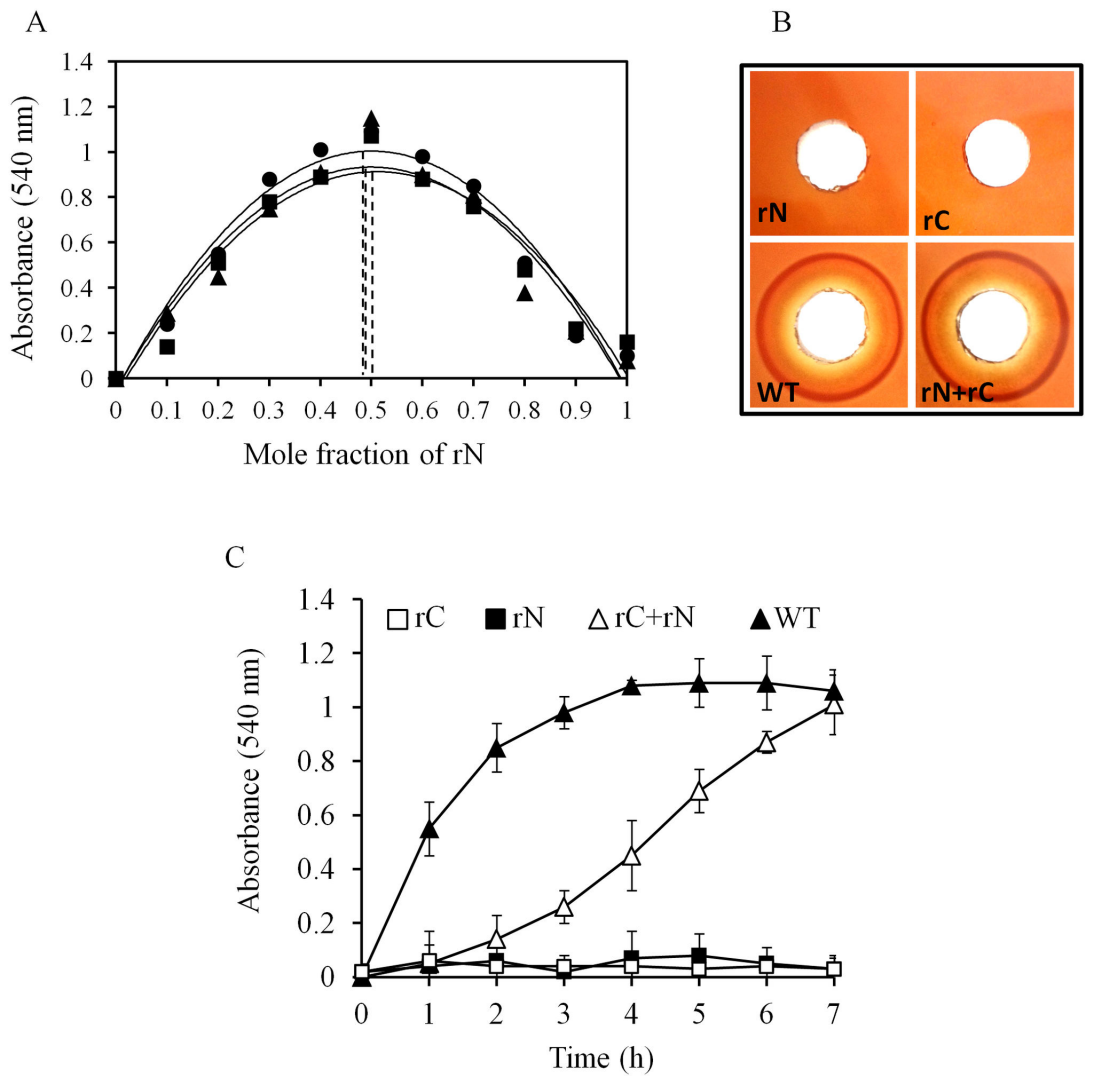
Characterization of haemolytic activity of N and C-domain mixture. (A) Job’s plot showing 1:1 complex formation for Cp-PLC rN and rC domain. Ten percent mouse erythrocytes were treated for 2h with varying molar fractions of N-domain with respect to C-domain keeping the total concentration constant (10 µM) and the absorbance of the mRBC supernatant was measured as a function of the molar fraction of N-domain on X axis. Results of three replicates were plotted separately. (B) Haemolysis assay for rN, rC, mixture of rN and rC and wild type Cp-PLC. All the protein mixes were spotted onto mouse blood agar plates and incubated at 37 °C for 4 h. (C) Kinetics of haemolysis. 5% mouse erythrocytes added with 10 micromoles each of rN, rC, mixture of rN and rC and wild type Cp-PLC proteins were incubated at 37 °C for 7 h. Absorbance of supernatants were recorded at 544 nm for every 1 h.

The observation that N and C-domains form a stable hybrid prompted us to determine the stoichiometry of the complex. To achieve this, we employed the continuous variation (Job's plot) method [23] and the restored hemolytic property of the hybrid was utilized as the parameter in this assay (Figure 5A). The plot depicted a symmetrical shape of the polynomial trend line and the maximum value of mole fraction of N-domain (~0.5) demonstrated the existence of N and C-domain complex at a 1:1 stoichiometrical ratio. We also evaluated the hemolytic activity on the blood agar plate with equimolar amounts of wild type and individual domains. The individual N and C-domains did not yield any hemolytic zone on mouse blood agar demonstrating their non-toxicity. The incubated mixture, on the other hand, generated zone of inhibition on par with wild type toxin (Figure 5B). To assess the kinetics of the Hemolysis, mouse erythrocyte suspensions were treated with wild type toxin and N and C-domain mixture in equimolar ratios and the absorbance of the supernatants was plotted as a function of incubation time. The N and C-domain hybrid lysed mRBCs but followed a delayed kinetics (Figure 5C) whereas the wild type toxin followed sigmoid kinetics. These results might suggest that the binding of N and C-domain hybrid to erythrocyte membranes may be delayed due to the minor shift/twist of C-domain in the hybrid, observed during prediction (Figure 4). Although delayed type, the unmistakable toxicity of N and C-domain hybrid directed us to look for the myonecrotic property *in vivo*.

### Cp-PLC C-domain complements the N-domain and restores the myonecrotic activity of the toxin *in vivo*

**Figure 6:**
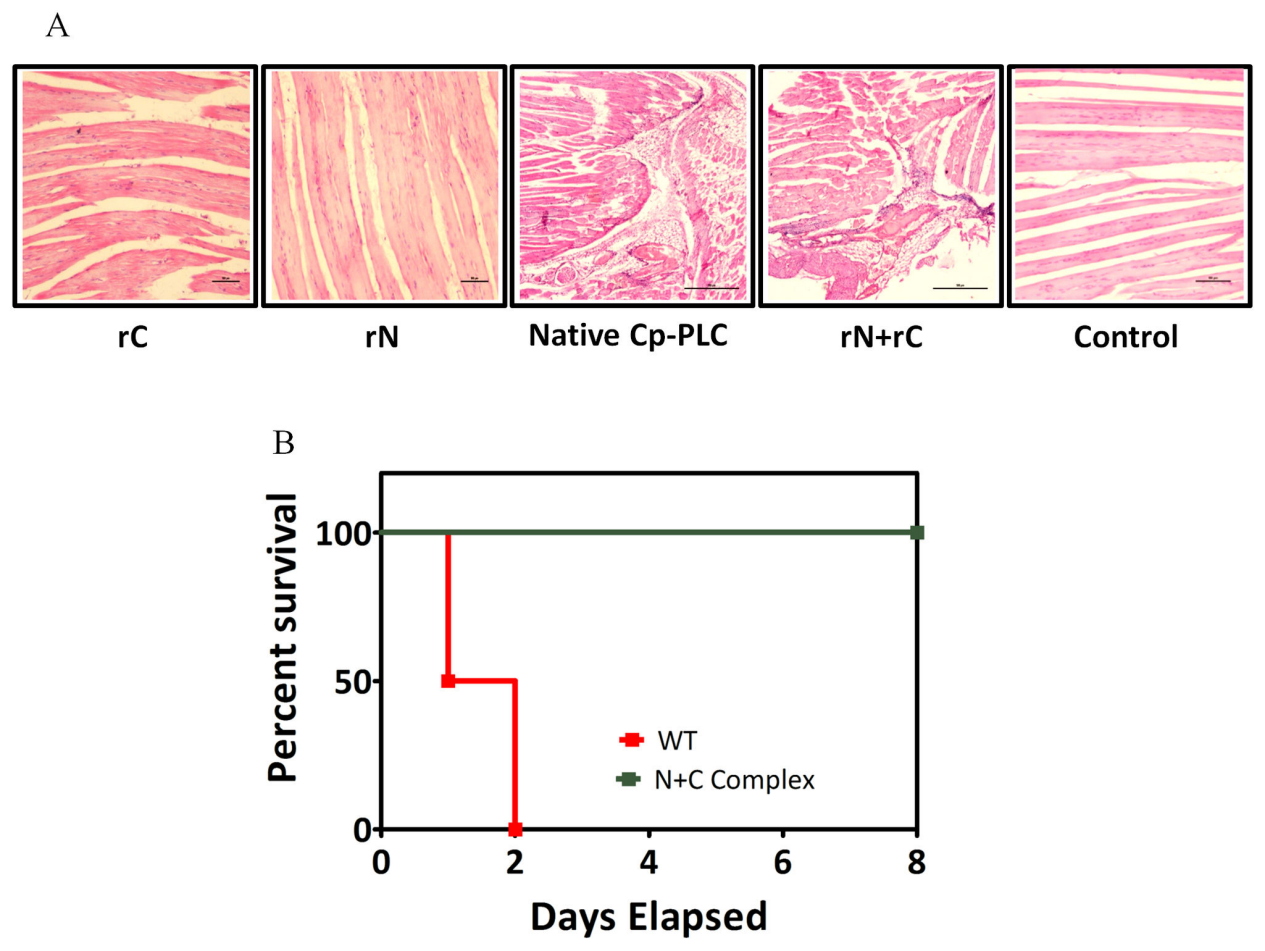
*In* *vivo* toxicity of N and C-domain mixture. (A) Mice challenged with rN+rC mixture and wild type toxin demonstrated severe necrosis of muscle tissues. (B) Kaplan-meier curves showing overall survival percentage of rN+rC mixture and wild type toxin injected mice.

To explore the functional relevance of the aforementioned binding, we assessed myonecrotic property of N and C-domain hybrid in comparison to wild type toxin *in vivo*. The results in Figure 6A show that muscle tissues from mice challenged with individual domains did not show any histopathological damage, whereas animals that received wild type or N and C-domain hybrid developed severe necrosis at the site of injection. Animals challenged with 10 H.U. native wild type toxin were found dead within 2 days (0% survival) whereas all the animals challenged with same amount of N and C-domain complex survived the observation period of 8 days despite myonecrosis (Figure 6B).

## Discussion

Since the discovery of Cp-PLC structure [6], many research groups have characterized the N and C-domains for their role in the biological function of the toxin by cloning and expressing the individual domains [10,11,22,24,25]. Three important observations from the previous studies led us to investigate the presence and thereby characterize the interactions between Cp-PLC N and C-domains. Firstly, it was reported that Cp-PLC exists in open and closed forms in the solution. The closed form of the toxin undergoes conformational changes when the C-domain binds to the host cell membranes resulting in the active cleft of N-domain getting access to the phospholipids [26]. The ten amino acid flexible linker (247-255 amino acids) between N and C-domains provides interim space for the conformational changes the two domains undergo after binding to host cells [6]. Therefore, it can be concluded that the flexible linker in Cp-PLC is essential to maintain the structural integrity of the protein and to keep the two domains intact. To our knowledge, no reports were found where mutations in the linker region affected haemolytic/cytolytic properties of Cp-PLC. A mutation in amino acid S250 to N250 did not affect the phospholipase and haemolytic activities of Cp-PLC [27]. Secondly, mutations at the amino acids presumed to be at the N and C-domain interfacial area of Cp-PLC did not affect the enzymatic activity of the toxin but reduced the haemolytic and cytolytic property [21,22]. Thirdly, it was demonstrated that distinct N and C-terminal domains when mixed can cause haemolysis [10,11]. Despite the fact that until now there is no evidence regarding the N and C-domain hybrid formation in the mixture, one might expect a functional intermediate considering the role of interfacial amino acids of the two domains in the toxicity. In our study, when individual recombinant N and C-domains without any linker amino acids were docked onto each other *in silico* and the structure of the complex is modelled, interactions between the two domains through at least six pairs of amino acid contacts were observed and no structural impediments were found on the active cleft of the N-domain. Previously, Naylor et al. [28] observed that two loops in N-domain move during the conversion of Cp-PLC from closed to open forms; 60-90 and 130-150 residues demonstrating their importance in toxicity. In our study, we observed that the interacting amino acids of N-domain involved residues in the 60-90 residue loop, thus placing the N-domain near to C-domain.

Furthermore, the findings of the current study showed that N-domain interacts with C-domain as demonstrated by *in vitro* pull down and far-Western blotting assays. Assuming that these interactions result in a stable N and C-domain hybrid, the question arises as to the possible functional relevance of this form of the alpha toxin. The ability of N and C-hybrid to cause hemolysis on blood agar plate as well as the release of haemoglobin in the supernatants of N and C-domain hybrid treated erythrocytes clearly demonstrated that the interactions restored the function [10, 11 and current study]. But on kinetic analysis, typically, wild type Cp-PLC toxin followed a pre-steady-state kinetics, and on the other hand, N and C-domain hybrid Cp-PLC resulted in sigmoid kinetics (Figure 5C) implicating the involvement of co-operative binding. Probably, the N and C-hybrid functions like a binary toxin (AB type toxin) where **b**inding C-domain docks onto cell membranes on host cells and the **a**ctive N-domain binds to membrane bound C-domain and causes cytolysis. One query still remains enigmatic, as to which of the two phenomena occur first; either binding of C-domain to membranes or N and C-domain binding. Though not a substantial evidence to derive at the mechanism, Titball et al. [11] showed sequential incubation of C-domain and N-domain did not confer haemolytic activity which might hint at formation of N and C-domain hybrid as primary step in the mechanism. 

As reviewed by Popoff M and Bouvet P [29], the catalytic N-domain of Cp-PLC is more likely derived from its enzyme precursors but the evolution of binding C-domain is still enigmatic, though it is found structurally identical to mammalian C2 domains belonging to Polycystin-1, Lipoxygenase, Alpha-Toxin (PLAT) superfamily. The catalytic property of *C. perfringens* N-domain, the structural similarity of C-domain with mammalian proteins, and currently, our elucidation of stable interactions between the N and C-domains might raise a potential concern regarding the safety of the N-domain *in vivo*, as it can bind to C2 domains of variety of membrane associated proteins (also belonging to PLAT superfamily) and lyse the host cells. The female BALB/c mice in our study, when challenged with N-domain alone did not show any observable histological abnormalities or necrosis (Figure 6). It is also well established that a structural and functional homolog of *C. perfringens* N-domain (the *Bacillus* phospholipase C enzyme) is non-hemolytic [10,30]. The reason for this non-cytotoxicity of the N-domain might be because the PLAT domain of mammalian structural proteins is located either transmembrane or intracellular and hence inaccessible for N-domain binding. 

In summary, we present *in silico*, *in vitro* and *in vivo* evidence implicating a stable interaction between the N and C-domains of *C. perfringens* alpha toxin and also the necessity of an unhindered active cleft on N-domain after interactions as a critical factor for the toxicity. 

## Supporting Information

Figure S1 **Superposition of Cp-PLC (PDB ID: 1CA1) and modelled Cp-PLC N and C-domain complex with N-domain in ribbon format and C-domain in stick format.** Cp-PLC N and C-domain residues are colored green and cyan, respectively and N and C-domains of the complex are colored pink and brown, respectively. The shift in orientation of C-domain is showed by arrow.
(PPTX)
