# Supplementary material for: In Silico, In Vitro and In Vivo Analysis of Binding Affinity between N and C-Domains of Clostridium perfringens Alpha Toxin
Source: PLoS One. 2013 Dec 11;8(12):e82024. doi: 10.1371/journal.pone.0082024 (PMC3859591; doi:10.1371/journal.pone.0082024)
Supplement: Figure S1 — Superposition of Cp-PLC (PDB ID: 1CA1) and modelled Cp-PLC N and C-domain complex with N-domain in ribbon format and C-domain in stick format. Cp-PLC N and C-domain residues are colored green and cyan, respectively and N and C-domains of the complex are colored pink and brown, respectively. The shift in orientation of C-domain is showed by arrow. (PPTX) [file pone.0082024.s001.pptx]

## Slide 1
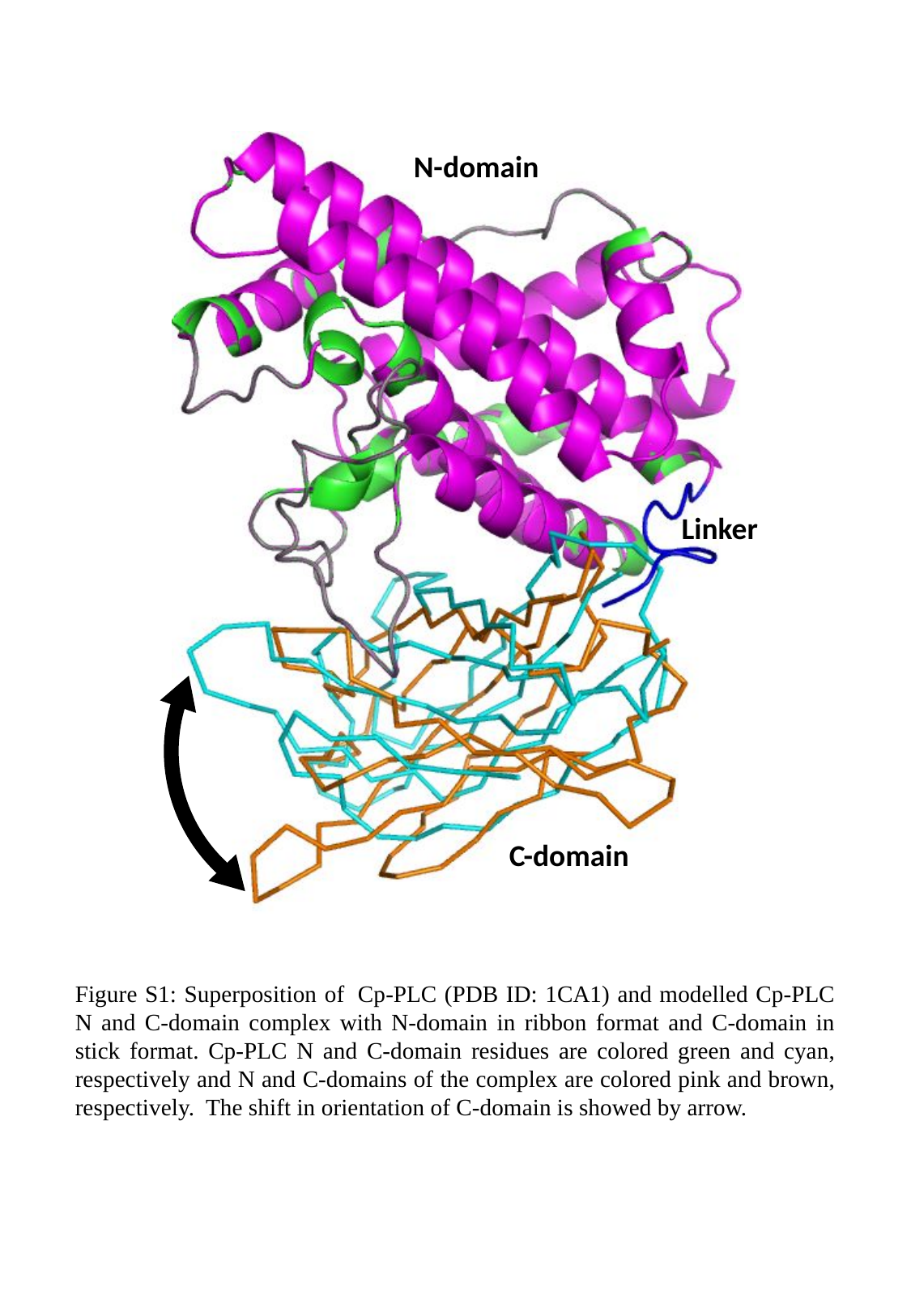

N-domain
Linker
C-domain
Figure S1: Superposition of  Cp-PLC (PDB ID: 1CA1) and modelled Cp-PLC N and C-domain complex with N-domain in ribbon format and C-domain in stick format. Cp-PLC N and C-domain residues are colored green and cyan, respectively and N and C-domains of the complex are colored pink and brown, respectively. The shift in orientation of C-domain is showed by arrow.
